# Identification of species-specific peptide markers in cold-pressed oils

**DOI:** 10.1038/s41598-020-76944-z

**Published:** 2020-11-17

**Authors:** Klaudia Kotecka-Majchrzak, Agata Sumara, Emilia Fornal, Magdalena Montowska

**Affiliations:** 1 Department of Meat Technology, Poznan University of Life Sciences, Wojska Polskiego 31, 60-624 Poznan, Poland; 2 Department of Pathophysiology, Medical University of Lublin, Jaczewskiego 8b, 20-090 Lublin, Poland

**Keywords:** Plant sciences, Biomarkers

## Abstract

In recent years, cold-pressed vegetable oils have become very popular on the global market. Therefore, new versatile methods with high sensitivity and specificity are needed to find and combat fraudulent practices. The objective of this study was to identify oilseed species-specific peptide markers, using proteomic techniques, for authentication of 10 cold-pressed oils. In total, over 380 proteins and 1050 peptides were detected in the samples. Among those peptides, 92 were found to be species-specific and unique to coconut, evening primrose, flax, hemp, milk thistle, nigella, pumpkin, rapeseed, sesame, and sunflower oilseed species. Most of the specific peptides were released from major seed storage proteins (11 globulins, 2S albumins), and oleosins. Additionally, the presence of allergenic proteins in the cold-pressed oils, including pumpkin Cuc ma 5, sunflower Hel a 3, and six sesame allergens (Ses i 1, Ses i 2, Ses i 3, Ses i 4, Ses i 6, and Ses i 7) was confirmed in this study. This study provides novel information on specific peptides that will help to monitor and verify the declared composition of cold-pressed oil as well as the presence of food allergens. This study can be useful in the era of widely used unlawful practices.

## Introduction

Growing consumption of cold-pressed plant oils and a wider range of available oil types have been observed on the market over the last few years. Currently, the most popular are olive oil, palm oil, rapeseed oil, soybean oil, and sunflower seed oil, but less common oils such as evening primrose seed oil, hemp seed oil, flaxseed oil, milk thistle seed oil, nigella seed oil, and pumpkin seed oil are gaining in importance. The global cold-pressed oil market value was $24.62 billion in 2018 and is expected to reach $36.40 billion by 2026, which is a 5.3% CAGR^1^. The reasons are the growing health awareness of the human population, increasing knowledge of the bioactive compounds detected in various oilseeds and their contribution to diet-related and skin disease prevention and treatment. Cold-pressed oils retain most of their bioactive and nutritional value since the oil’s temperature during the pressing process lies below 50 °C. Therefore, for health benefits, nutritionists advise regular diet supplementation with small amounts (1–3 tablespoons per day) of cold-pressed oils, due to higher yields of pro-healthy compounds over refined oils. They may regulate the blood lipid profile and insulin level, as well as offer anticancer and antioxidant activity^2,3^. Another trend observed is the modification of food products and nutraceuticals to improve their nutritional composition and positive impact on human health through the addition of edible oils^3,4^. In particular, in animal food products, the partial replacement of the fat or meat fraction with a plant oil/protein fraction is more frequent^4,5^.

Oilseeds are rich in *n*-3 and *n*-6 essential fatty acids, protein, carbohydrate, fibre, vitamins, minerals, and many small bioactive components belonging to different classes, including phytosterols, tocopherols, phenolic compounds, flavonoids, carotenoids, and bioactive peptides. For instance, hemp oil has a high content of antioxidants, such as phenolic compounds, tocopherols, and phytosterols as well as flavonoids, including flavanones, flavanols, flavonols and isoflavones. Milk thistle oil is a source of silymarin and mixtures of various flavonolignans, i.e. silydianin, silychristin, silybin, and isosilybin, and flaxseed oil is rich in phenols, flavonoids, α- and δ -tocotrienol, and carotenoids, including lutein and β-carotene^6–8^. The presence of proteins and peptides in cold-pressed oils is less commonly discussed, though they are an important component that may influence the oils’ stability and also cause an allergenic response in sensitive consumers^2,3^.

The use of cold-pressed oil by the food industry is anticipated to reach the highest growth rate in the next few years. The oils’ online retail distribution channel is also expected to grow considerably^1^. Since unprocessed oils reach high prices and are a desirable commodity on the market, they are products susceptible to counterfeiting^9,10^. Therefore, there is an urgent need to implement modern and sophisticated analytical methods to detect adulterations of high-price cold-pressed oils derived from various oleaginous seeds and fruits. Many advanced untargeted approaches have been introduced recently to address authenticity issues of edible oils, based on liquid or gas chromatography mass spectrometry (LC–MS or GC–MS) techniques for measuring various polar, nonpolar, and volatile compounds, as well as spectroscopic techniques including nuclear magnetic resonance (NMR) spectroscopy, near-infrared spectroscopy (NIR), attenuated total reflection-Fourier transform infrared spectroscopy (ATR-FTIR), or fluorescence, where the result is achieved by analysing the whole spectrum of oils and development of statistical models^11–14^.

When food fraud is considered in terms of food safety issues, and related, for example, to food allergenicity, a targeted approach based on identifying specific proteins and peptide markers could be a method of choice. From this aspect, MS-based proteomic analysis has the advantage of detecting the adulteration of unrefined oils and tracing potential allergenic proteins present in the sample, simultaneously during one MS run. Depending on the type of oil, the method of protein extraction and assessment methodology, the protein content of edible oils varies from 0.01 to 14.8 ppm^2,15^. Allergenic proteins, such as cruciferin (11S globulin) and napin (2S albumin) have been identified in rapeseeds (*Brassica napus*) but also in unrefined rapeseed oil using a gel-based LC–MS method; however, these allergens have not been shown in refined rapeseed oil^16^. Moneret-Vautrin et al.^17^ found that the residual allergenic proteins, i.e. Ara h1, in peanut oil can cause an allergic reaction in infants with atopic dermatitis sensitive to peanut, whereas, Blom et al.^18^ estimated the risk of allergenic reactions to refined peanut oil as extremely low.

This study aimed to identify the unique peptide markers specific to oilseed species and proteins present in cold-pressed oils using proteomic techniques. An additional goal was to assess the presence of allergens. The protein and peptide composition of oils produced from 10 oilseeds, namely coconut, evening primrose, hemp, flax, milk thistle, nigella (also known as black cumin), pumpkin, rapeseed, sesame, and sunflower seeds, was analysed using ultra-performance liquid chromatography coupled to quadrupole time-of-flight mass spectrometry (UHPLC-Q-TOF–MS/MS). The markers identified could be useful for the detection of adulteration.

## Results and discussion

### Protein profiles

**Figure 1:**
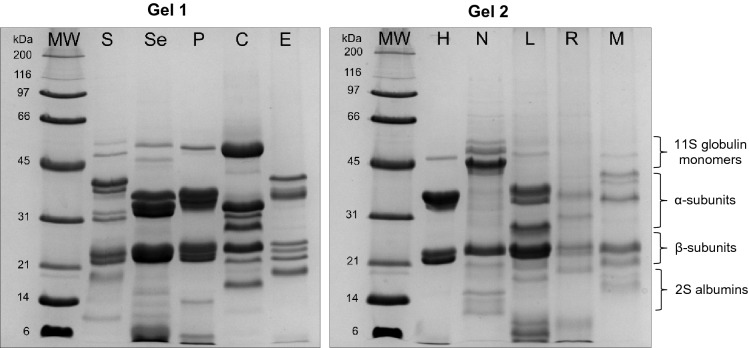
Protein profiles of 10 acetone-extracted cold-pressed oils. Lanes: S—sunflower; Se—sesame; P—pumpkin; C—coconut; E—evening primrose; H—hemp; N—nigella; L—flax (linen); R—rapeseed; M—milk thistle.

A previous study, comparing five methods using various solvents to separate proteins from unrefined and refined oils, showed that the protein content differs significantly depending on the extraction method^15^. Therefore, the two most efficient methods were tested on cold-pressed sunflower oil, i.e. extraction with acetone-hexane and extraction with acetone, at the beginning of this work. Since no considerable differences were observed in the extracted protein profiles, extraction with acetone was applied to all oil samples. Acetone extraction has been used successfully to identify allergenic proteins from commercial cold-pressed rapeseed oils^16^. Proteins were present in all the examined oils but the proteomic profiles differed considerably in the distribution and intensity of protein bands (Fig. 1). Visible differences reflect the species diversity of the major storage proteins. Under reducing SDS-PAGE conditions, the most abundant bands belonged to 11S globulin α and β subunits (MW 30–45 and 20–30 kDa, respectively). 11S globulin monomers (MW about 45–56 kDa) were less intense. Lower molecular weight bands (MW 10–21 kDa) were less abundant but showed species specificity.

Globulin, albumin and oleosin subunits have been reported in electrophoretic bands obtained from oilseed meals or isolates of various species, such as sesame protein isolate^19^, hemp protein isolate^20^ and sunflower seed and kernel proteins^21^. Regarding cold-pressed oils, proteins are minor components present in very low amounts as a result of gentle processing by pressing the oilseeds with a screw press without heating, and the resulting oil is purified only by sedimentation, filtration, or centrifugation. Therefore, identification of the proteins in vegetable oils has not been common in the literature. The fruit and seed proteins, mainly storage proteins and different oleosins have been found in olive oil and palm oil^22,23^, whereas knowledge about nigella, milk thistle, and evening primrose proteins is negligible. Major rapeseed allergens, such as napin and cruciferin (2S albumin and 11S globulin, respectively), have been extracted and identified from commercial cold-pressed rapeseed oil^16^. Accordingly, there is a need to conduct research to determine species-specific proteins and peptides found in cold-pressed vegetable oils.

### Protein identification

**Figure 2:**
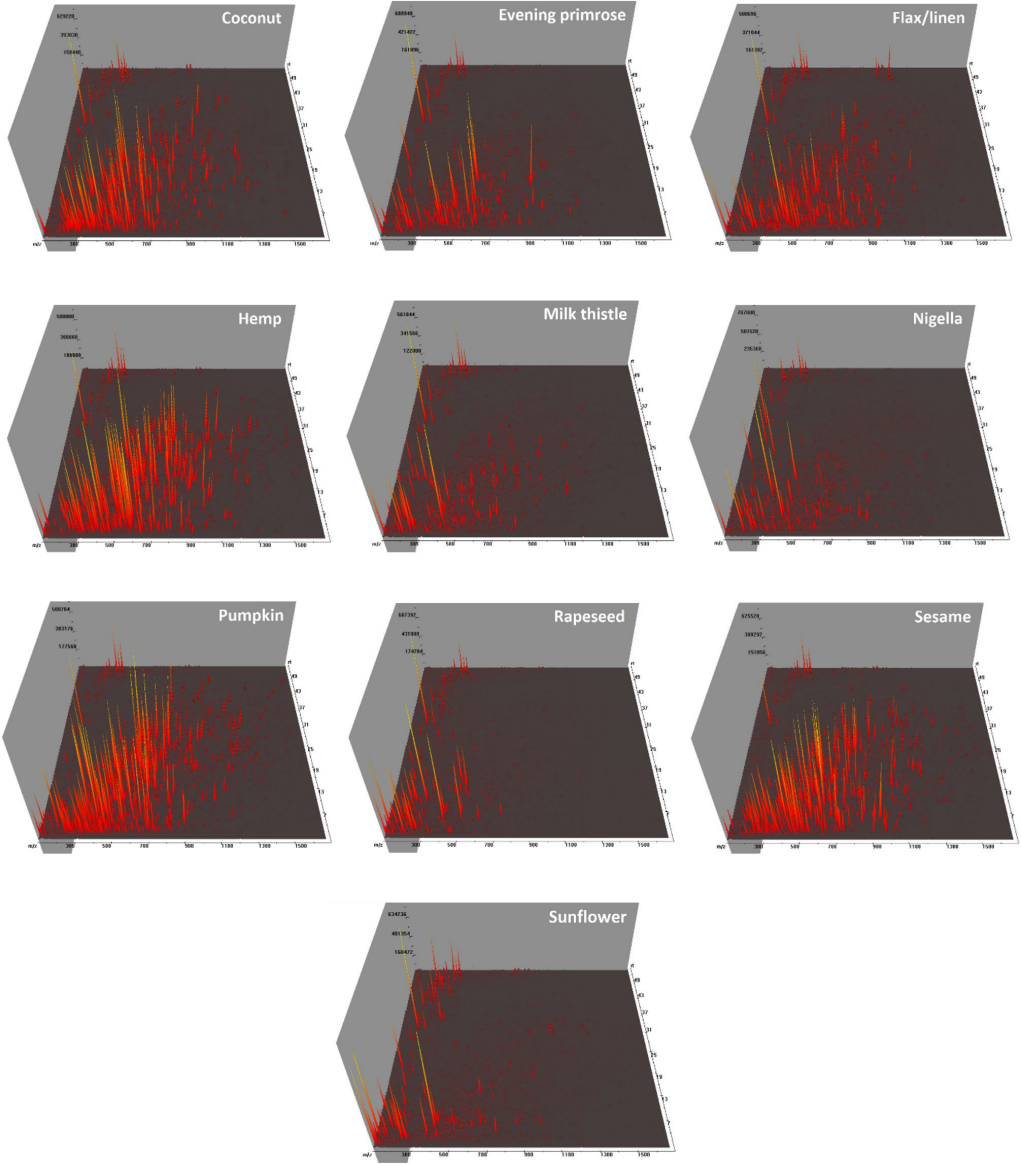
3D LC-Q-TOF–MS/MS chromatograms of tryptic digested proteins extracted from cold-pressed oils obtained from 10 species of oilseeds: coconut, evening primrose, flax/linen, hemp, milk thistle, nigella, pumpkin, rapeseed, sesame, and sunflower. The mass-to-charge ratio (*m/z*), the retention time, and the intensity of the signal are present on the X-, Y-, and Z-axis, respectively.

**Figure 3:**
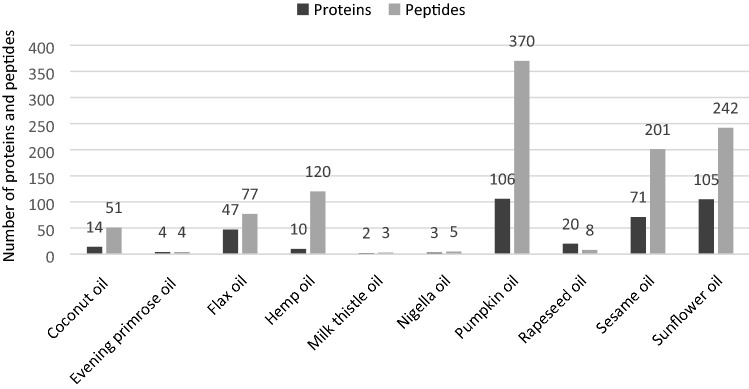
Number of identified proteins and tryptic peptides obtained from cold-pressed oils.

To identify specific proteins, an in-solution tryptic digest of acetone-extracted proteins was analysed using the UHPLC-Q-TOF–MS/MS method. Figure 2 presents the 3D chromatograms obtained from the 10 examined cold-pressed oils. The National Center for Biotechnology Information (NCBI, U.S. National Library of Medicine) protein database was searched for protein and peptide identification across green plant taxonomy entries or appropriate genera (i.e. *Brassica*, *Cannabis*, *Cocos*, *Cucurbita*, *Helianthus*, *Nigella*, *Linum*, *Oenothera*, *Sesamum*, and *Silybum*). The number of identified proteins and tryptic peptides obtained from cold-pressed oils is shown in Fig. 3. In total, over 380 proteins and 1050 peptides were identified in the samples analysed. The highest number of proteins and peptides was found in the pumpkin and sunflower oils (106 and 105 proteins, respectively). The smallest number of proteins and peptides was determined in evening primrose, milk thistle, and nigella oils (only four, two, and three proteins, respectively). For these species, only a small fraction of the proteins have been sequenced, and thus, databases are highly incomplete. SDS-PAGE protein profiles obtained from these three oils confirmed the presence of a storage protein bands of species-specific distribution and intensity (Fig. 1).

All proteins which were identified with high confidence scores using the Spectrum Mill MS Proteomics Workbench with > 70% score peak intensity and 10 ppm precursor mass tolerance are gathered in Supplementary material 1 for protein datasets. The matches and Spectrum Mill scores were evaluated at 1% of the false discovery rate (FDR), for identity and homology threshold. The identified proteins belong, among others, to 11S and 7S globulins, 2S albumins, and oleosin families. Some of those derived from hemp oil and sesame oil have been identified previously in hempseed and sesame seed defatted powders using LC–MS/MS^24,25^. For the remaining eight oilseed species studied, there is a lack of comprehensive proteomic MS-based analysis. Recently, only the key proteins of oil palm mesocarp (*Elaeis guineensis*) have been identified by 2-DE and MALDI-TOF/TOF methods^23^. None of them coincided with the proteins detected in coconut oil, despite the close relationship between these two species as they belong to the same family of Arecaceae.

### Species-specific peptide markers

The peptides identified and selected, based on the Spectrum Mill output scores, were searched against the NCBInr database using the protein Basic Local Alignment Search Tool (BLAST) and blastp algorithm (U.S. National Library of Medicine, Bethesda, MD), for species and protein specificity. In total, 92 species-specific peptides, that were released from 42 proteins (including different subunits), were identified in the examined oils. The largest amount of unique peptides was found in sesame, sunflower and pumpkin oils (21, 18, and 17 peptides, respectively). Table 1 presents the unique, species-specific peptide markers identified in coconut, evening primrose, flax, hemp, milk thistle, nigella, pumpkin, rapeseed, sesame, and sunflower cold-pressed oils. The peptides’ total intensities were in the range of 10^5^–10^8^ and scored peak intensities (SPI) were > 60%. All peptides detected in the examined cold-pressed oils are shown in Supplementary material 2 for peptide datasets.

**Table 1:** Unique peptide markers identified in coconut, evening primrose, flax, hemp, milk thistle, nigella, pumpkin, rapeseed, sesame, and sunflower cold-pressed oils.

Protein	Protein score	Parent ion (m/z)	Mr (exp)	Exp z	Total intensity	RT (min)	SPI (%)	Delta (ppm)	Peptide marker
**Coconut**									
11S globulin isoform 2 (AKS26849.1)	234.02	635.3332	1269.6474	2	2.28E+07	20.45	94.1	9.3	AGSEGFQFVSIK
		991.5129	1982.0052	2	3.44E+06	40.52	91.7	6.8	GFGTELLAAAFGIDMELAR
		714.3443	2140.9977	3	5.77E+06	27.08	83.8	9.7	GMVGLVMPGCPETFQSFQR
Cocosin (ASQ40963.1)	202.8	929.0134	929.0134	2	1.06E+07	29.65	94.1	7.7	QGQLLIVPQNFAMLER
		1011.4302	2021.8359	2	1.13E+07	19.43	88.0	8.5	SEAGVTDYFDEDNEQFR
Oleosin isoform OLE500c partial (ACH91013.1)	16.77	599.3204	1197.6222	2	3.04E+06	10.67	78.7	9.4	VPGAEQLEQAR
**Evening primrose**									
Putative LOV domain-containing protein (AML79398.1)	2.42	575.2869	1723.8511	3	1.03E+06	15.48	41.5	− 2.8	DPGSRLGSNNGSXFFR
**Flax**									
Conlinin (CAC94011.1)	133.74	658.3132	1315.6059	2	5.62E+06	6.47	96.3	10.0	DLPGQCGTQPSR
		745.3305	1489.6448	2	3.53E+06	1.40	79.9	6.0	GGQGGQGQQQQCEK
		857.4170	1713.8151	2	4.60E+07	5.05	92.4	6.8	QDIQQQGQQQEVER
Conlinin (CAC94010.1)	101.34	1331.6097	2662.2023	2	2.19E+07	14.87	73.1	3.7	GGGQGGQGQQQSCEQQIQQQDFLR
		863.9330	1726.8467	2	5.02E+06	4.45	93.4	6.9	QEIQQQGQQQEVQR
Oleosin high molecular weight isoform (ABB01624.1)	50.01	600.2677	1199.5184	2	1.29E+06	8.17	80.4	8.2	MQDAAGYMGQK
		1082.5430	2164.0669	2	1.35E+07	19.22	83.9	5.4	YLQQAGQGVGVGVPDSFDQAK
Oleosin high molecular weight isoform (ABB01616.1)	51.29	1089.5514	2178.0826	2	7.14E+06	19.38	94.4	6.0	YLQQAGQGVGVGVPDSFEQAK
Oleosin low molecular weight isoform, partial (ABB01620.1)	24.57	795.8659	1590.7143	2	3.35E+06	6.60	62.5	6.5	ASDFGQQHVTGQQTS
Oleosin low molecular weight isoform (ABB01617.1)	15.34	838.3924	1675.7671	2	8.05E+06	7.17	75.4	6.2	ASEFAQQHVTGGQQTS
Oleosin low molecular weight isoform (ABB01618.1)	14.42	831.3859	1661.7514	2	1.54E+07	6.78	66.3	7.8	ASEFGQQHVTGGQQTS
**Hemp**									
Edestin 3 (SNQ45160.1)	453.22	847.4206	1693.8214	2	6.73E+07	19.87	94.8	7.4	AMPEDVIANSYQISR
		889.4256	1777.8293	2	1.81E+07	15.58	94.4	8.2	FYIAGNPHEDFPQSR
		1049.0236	2097.0247	2	1.54E+08	30.92	82.7	7.3	GFSVNLIQEAFNVDSETAR
		846.7562	2538.2293	3	1.04E+08	29.63	93.6	9.8	GVLGTLFPGCAETFEEAQVSVGGGR
		1166.5912	2332.1655	2	4.89E+06	21.75	66.8	4.2	NAMYAPHYNINAHSIIYAIR
		834.3816	1667.7442	2	2.15E+07	8.68	92.9	7.0	TAVYGDQNECQLNR
		1044.4852	3131.4132	3	2.41E+07	25.43	95.7	8.9	VECEGGMIESWNPNHEQFQCAGVALLR
Edestin 3 (SNQ45158.1)	390.94	823.0533	2467.1282	3	6.42E+06	20.03	88.8	7.0	FYIAGNPHQEFPQSMMTQQGR
		563.7565	1126.4946	2	2.08E+06	3.43	75.7	9.9	LEACEPDHR
		800.4523	1599.8853	2	1.34E+07	20.57	83.6	7.5	QGQALTVPQNFAVVK
7S vicilin-like protein (SNQ45153.2)	65.5	1016.029	2031.0393	2	1.53E+06	22.47	74.8	5.6	EILSSQQEGPIVYIPDSR
**Milk thistle**									
Preprosilpepsin 2 (AGE15495.1)	38.35	764.4145	1527.8094	2	6.24E+06	28.93	94.9	8.1	IFELTPEQYIFK
		772.3684	2315.0687	3	1.67E+07	21.68	78.4	9.5	NVNEEEGGELVFGGVDPNHFR
**Nigella**									
Chain A, nigellin-1.1 (pdb|2NB2|A)	51.07	775.3724	1549.7284	2	1.08E+06	18.88	85.7	5.9	ACIGLCAPACLTSR
		673.8030	1346.5868	2	2.54E+06	17.22	94.1	8.8	CTYIPDYAGMR
		716.2902	1431.5627	2	2.23E+06	6.40	72.0	7.2	YQDCLSECNSR
Thionin NsW1 (C0HJH9.1)	11.29	521.7133	1042.4114	2	1.55E+06	3.47	53.8	7.5	TCSGLCGCK
**Pumpkin**									
11S globulin subunit beta-like (XP_023553731.1)	552.27	1039.4212	2077.8217	2	5.40E+06	9.22	95.5	6.5	GDEQQWEEEQEEEQER
		1215.0627	2429.1038	2	9.60E+06	17.10	95.0	5.9	GVVLSGCPETYQESQQSAGEFR
		805.1246	2413.335	3	1.77E+07	31.4	94.0	10.0	LIIVVLLDVSNHANQLDFHPR
		699.8968	1398.7740	2	4.62E+07	14.53	90.0	8.8	VEGQFEVIKPPR
		786.9318	1572.8414	2	6.18E+07	23.20	98.1	9.5	VLAEVLNINTEMAR
Vicilin-like (XP_023527143.1)	408.19	885.8216	2655.4287	3	5.85E+07	26.48	96.6	8.2	LSEGGVLVIPAGHPIAIMASPNENLR
		709.3693	1417.7183	2	1.09E+07	16.30	93.0	9.3	LVGFGINAENNNR
		671.3671	1341.7161	2	1.82E+06	13.52	90.0	8.1	LVQPVNNPGEFK
		1030.9556	2060.8944	2	6.23E+06	11.92	84.9	4.6	TEQEQSNNPYYFQEQR
11-beta-hydroxysteroid dehydrogenase 1B-like (XP_022923933.1)	133.1	878.9664	1756.9150	2	1.17E+06	25.58	83.0	6.0	DALIGLVPVETAEACAK
		823.4332	1645.8465	2	1.91E+06	18.43	92.8	7.7	ILAMPAAGASESDALTK
		788.9426	1576.8668	2	2.94E+06	26.02	88.7	7.0	IVALSAPPAWMPAPR
2S albumin-like (XP_023520423.1)	74.43	669.3239	2005.9396	3	5.74E+05	15.18	67.2	8.8	AHEEIGSCVQYLTQQSR
11 kDa late embryogenesis abundant protein-like (XP_022940345.1)	63.21	823.9135	1646.8093	2	4.18E+06	4.53	71.4	6.4	TGAHTGLTTGTGTGTGTR
oleosin 18.2 kDa-like (XP_022928435.1)	62.5	632.8148	1264.6128	2	2.82E+06	2.40	73.5	7.6	TTTTTAAQEQGR
17.1 kDa class II heat shock protein-like (XP_022982419.1)	21.0	800.4250	1599.8337	2	6.42E+05	15.97	94.0	5.7	VQIEDNQLVVTGER
17.4 kDa class I heat shock protein-like (XP_022939316.1)	14.44	766.3943	1531.7711	2	2.40E+06	8.95	64.0	6.7	VPSSGAGETTAIANTR
**Rapeseed**									
Napin small chain S2 (pir||S70337)	14.45	537.2426	1073.4680	2	3.14E+06	4.98	86.1	9.2	QAMQPGGGSGPS
**Sesame**									
Legumin B-like (XP_020549903.1)	421.56	1398.1465	2795.2689	2	5.79E+07	29.35	97.2	6.0	AGEQGCEWVEFNTNDNALINTLSGR
		1113.6036	2226.1852	2	1.09E+08	26.83	87.0	6.6	ALMLPAYHNAPILAYVQQGR
		764.7779	2292.2962	3	7.37E+07	44.67	95.1	10.0	FSTINSLTLPILSFLQLSAAR
		691.9919	2073.9407	3	7.24E+07	14.87	85.1	9.9	GESDMQIVNHNGQAVFDGR
		1055.5338	2110.0451	2	3.88E+07	34.67	96.6	7.2	GFDVQILSEVFGVDEQTAR
		794.4355	1587.8489	2	9.07E+07	23.93	97.2	9.3	GLPADVIANAYQISR
		952.0832	2854.2117	3	2.71E+07	23.70	94.1	8.2	GMYGVMISGCPETFESSQQQFEEGR
		752.3698	1503.7186	2	3.14E+07	10.37	91.7	9.1	GQEQQEYAPQLGR
		595.3016	1189.5861	2	2.32E+07	10.82	64.3	8.3	GQHQFGNVFR
		604.2730	1207.5273	2	7.92E+06	4.22	66.6	9.4	GSTWQQGQCR
		1025.8021	3075.3650	3	1.58E+07	21.77	89.4	8.7	IQAEGGVSEFWDHNSDEFQCAGVSIHR
7S globulin (AAK15089.1)	154.07	614.7641	1228.5124	2	6.43E+05	1.18	60.1	7.0	GCEQQHGEQR
		934.9765	1868.9330	2	3.40E+06	26.42	71.1	6.8	IPYVFEDQHFITGFR
		982.1603	2944.4404	3	1.49E+07	37.47	100.0	8.8	LLQPVSTPGEFELFFGAGGENPESFFK
		1003.8700	3009.5720	3	2.31E+06	31.63	84.5	7.8	VAILEAEPQTFIVPNHWDAESVVFVAK
Steroleosin (AAL09328.1)/11-beta-hydroxysteroid dehydrogenase 1B-like (NP_001291322.1)	82.77	578.3271	1155.6368	2	6.91E+05	17.05	87.6	8.8	DLGSPDVVVVR
		614.8542	1228.6896	2	1.31E+06	16.68	75.7	9.5	DVQVSTTPILR
		800.4400	1599.8629	2	1.47E+06	21.57	94.7	6.2	SLLYPETVQVPEPK
2S albumin precursor isoform 3 (ABB60053.1)	68.82	655.7865	1310.5538	2	1.96E+07	9.13	96.1	9.1	EQEMQQMMQK
		695.7788	1390.5371	2	6.28E+06	10.52	79.1	9.5	MCGMSYPTQCR
Caleosin (AAF13743.1)/Peroxygenase (NP_001291323.1)	55.71	697.0523	2089.1223	3	1.41E+06	20.25	87.5	9.6	NAALAPDAPLAPVTMERPVR
		751.8763	1502.7348	2	1.82E+06	30.18	87.0	7.0	YLPMNFENLFSK
**Sunflower**									
11S globulin seed storage protein G3-like (XP_021988017.1)	234.0	973.9860	1946.9566	2	1.24E+07	19.9	94.3	4.1	VQIVDNQGNSVFDNELR
11S globulin seed storage protein G3-like (XP_021973262.1)	172.37	704.4060	2111.1832	3	2.76E+06	19.38	82.2	9.6	GHIVNVGQDLQIIRPPQAR
		973.4946	1945.9726	2	1.89E+06	19.23	93.9	4.8	VQIVNNQGNSVFDNELR
Putative 11-S seed storage protein (OTG28570.1)	209.52	581.7473	1162.4793	2	1.35E+06	1.53	75.8	6.9	GMDSSADSHQK
		885.4581	2654.3420	3	3.28E+06	29.82	80.9	6.7	NQEVVAIIVDDVNNPANQLDFQAK
		610.8050	1220.5906	2	8.31E+06	12.27	91.1	9.9	SPFGGQEELTR
2S seed storage albumin 1 (XP_021993221.1)	168.15	655.7993	1310.5794	2	4.32E+06	8.80	87.1	9.2	GQFGGQEMETAR
2S seed storage albumin 2 precursor (XP_021993622.1)	153.25	654.8089	1308.6001	2	1.37E+07	13.68	90.9	8.0	GQFGGQEMDIAR
11S globulin subunit beta-like (XP_021998813.1)	123.79	700.0163	2098.0173	3	9.00E+06	5.32	52.0	8.1	TGQSQRPGWETGRPEQQR
Oleosin 16.4 kDa-like (XP_022005568.1)	72.45	736.8887	1472.7591	2	6.82E+06	10.92	76.8	7.5	QITGTVPEQVDSAK
Vicilin-like seed storage protein At2g28490 (XP_022001204.1)	60.64	1050.9588	2100.9033	2	1.49E+06	23.27	90.1	3.4	NDYGWSVEVDGDDYEPLK
Jacalin-related lectin 34-like (XP_022000240.1)	55.76	790.3864	2369.1229	3	2.82E+06	13.10	71.4	9.2	GTGTGTFGTGGHEGLGTNIGHVEGR
		897.7587	2691.2427	3	5.97E+05	15.87	63.1	6.9	SIGQTGSQGLGTEPGSHGGIMGDYGATR
		792.8624	1584.7071	2	1.54E+06	11.05	81.4	6.6	TGGIMGEYGSTGQGNR
Oleosin, partial (CAA44224.1)	48.65	669.3257	1337.6332	2	4.42E+06	8.73	98.2	8.1	GTLQDAGEYAGQK
		747.3840	1493.7482	2	2.11E+07	15.60	71.9	8.3	QTAGSVPESLDYVK
Oleosin (CAA55348.1)	31.67	684.3487	1367.6815	2	1.83E+06	2.62	73.7	6.3	HHVTTTQPQYR
		619.3114	1237.6059	2	2.07E+06	8.98	89.5	7.8	LQDVGEYTGQK

**Figure 4:**
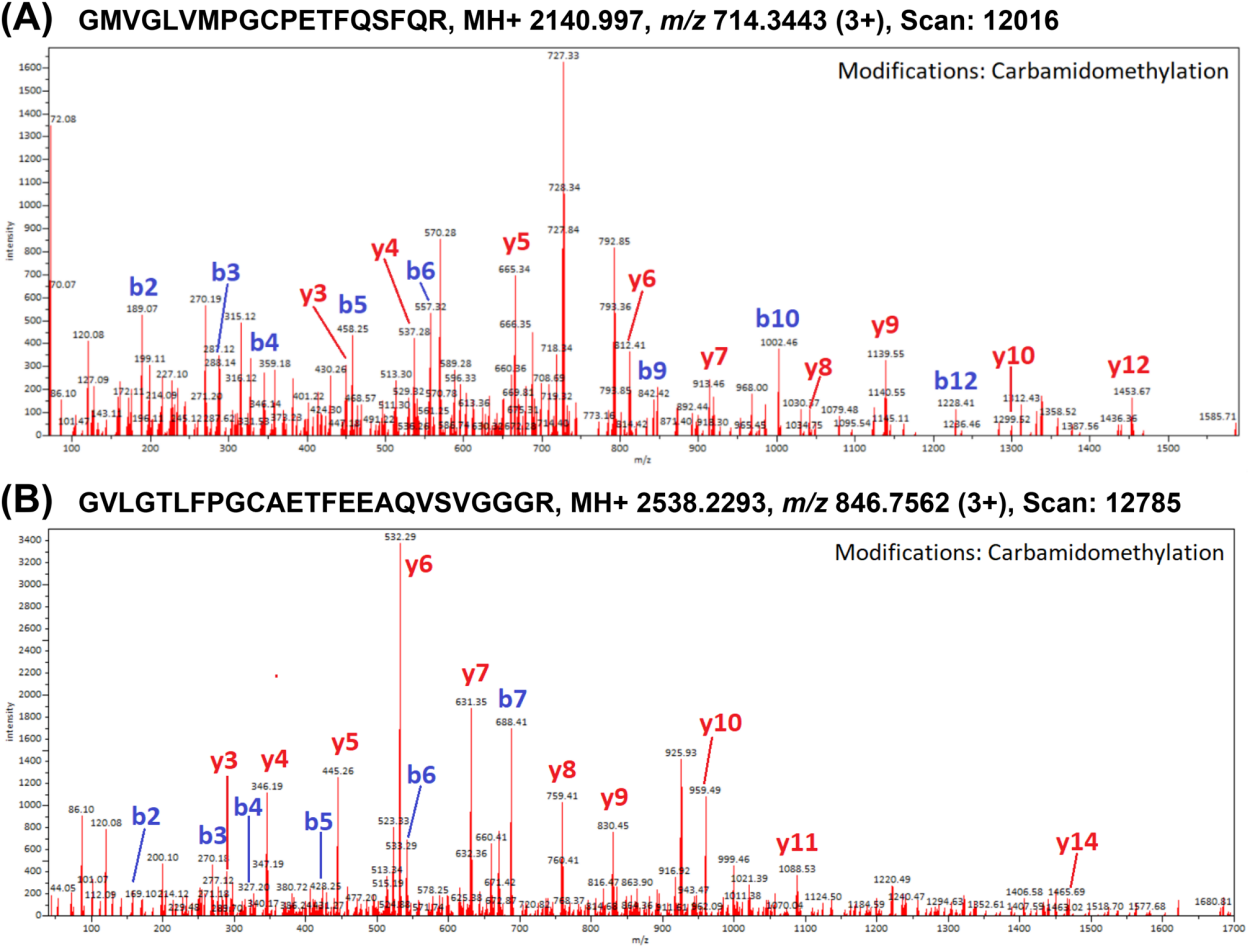
Mass spectra of peptide markers specific to coconut and hempseed obtained from 11S globulins extracted from: (**A**) coconut oil (11S globulin isoform 2, AKS26849.1); (**B**) hemp oil (edestin 3, SNQ45160.1).

**Figure 5:**
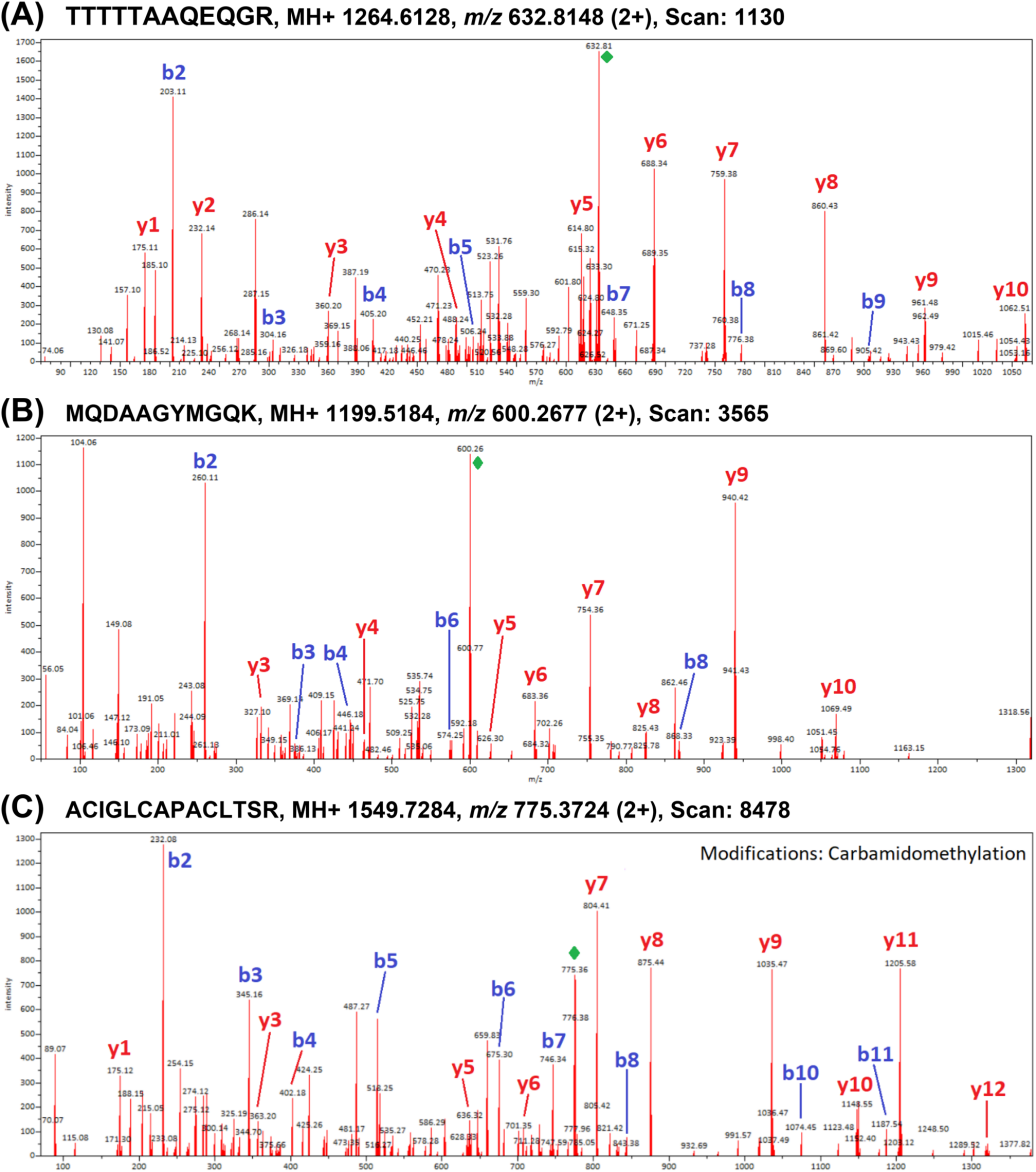
Mass spectra of species-specific peptide markers obtained from proteins extracted from cold-pressed oils. (**A**) Pumpkin oleosin 18.2 kDa-like (XP_022928435.1); (**B**) flax oleosin high molecular weight isoform (ABB01624.1); (**C**) Nigella nigellin-1.1 chain A (pdb|2NB2|A).

Unique peptides derived mainly from major seed storage proteins (i.e. cocosin, conlinin, edestin, other specific legumin-like or vicilin-like globulin subunits, and 2S albumins), and oleosins which are structural proteins found in plant parts with high oil content. Mass spectra of two peptide markers specific to coconut and hempseed obtained from 11S globulins are shown in Fig. 4. Product spectra of another three peptides unique to pumpkin oleosin 18.2 kDa-like, flax oleosin high molecular weight isoform, and black cumin nigellin-1.1 chain A, are shown in Fig. 5. In the present study, most of the identified and selected peptides were specific to the species investigated, but in some cases the peptides were assigned to another species of a given genus, i.e. *N. damascena* or *C. moschata*, and thus, the specificity of the protein for the genus can be confirmed.

To date, only a few comprehensive proteomic studies of oilseeds having application to food science have been published. Many protein sequences submitted to the NCBI database were obtained by methods of DNA sequencing. Several peptides released from hemp edestin 1 and edestin 2 identified in our study (Supplementary material 2) have been reported previously in hempseed defatted flour^24^.

Regarding allergens with food route exposure, proteins derived from four of the ten species investigated, namely pumpkin, rapeseed, sesame, and sunflower, have been included in the database of officially recognized allergens maintained by the World Health Organization and International Union of Immunological Societies (WHO/IUIS) Allergen Nomenclature Sub-committee^25^. In the present study, some food allergens derived from pumpkin, sesame, and sunflower cold-pressed oils were detected. These allergens include pumpkin Cuc ma 5, sunflower Hel a 3, and six sesame allergens (Ses i 1, Ses i 2, Ses i 3, Ses i 4, Ses i 6, and Ses i 7). Details of the allergens and detected peptides are shown in Table 2. Recently, seven sesame allergens have been detected in raw sesame seeds and quantified in food products such as sauces, cookies, cake, and candy^26^ based on selected signature peptides. Eight out of twelve of those previously selected signature peptides for sesame allergens were identified in the present study.

**Table 2:** Allergens identified in cold-pressed oils.

Species	Allergen name/isoallergen	Protein	Accession no	Peptide sequence
Pumpkin	Cuc ma 5/Cuc ma 5.0101	2S albumin	Q39649.1	CDMLEEIAREEQR
				EGGSFDECCR
				NLPSMCGIRPQR
				NVDEECRCDMLEEIAR
Sunflower	Hel a 3/Hel a 3.0101	Putative lipid transfer protein	AAP47226.1	VRPDMASSLPGK
Sesame	Ses i 1/Ses i 1.0101	2S albumin	AAK15088.1	QQQQEGGYQEGQSQQVYQR
	Ses i 2/Ses i 2.0101	2S albumin	AAD42943.1	MCGMSYPTECR
	Ses i 3/Ses i 3.0101	7S vicilin-like globulin	AAK15089.1	IPYVFEDQHFITGFR
	Ses i 3/Ses i 3.0101	7S vicilin-like globulin	AAK15089.1	SFSDEILEAAFNTR
	Ses i 4/Ses i 4.0101	Oleosin	AAG23840.1	GVQEGTLYVGEK
	Ses i 6/Ses i 6.0101	11S globulin	AAD42944.1	IQSEGGTTELWDER
	Ses i 7/Ses i 7.0101	11S globulin	AAK15087.1	FESEAGLTEFWDR
	Ses i 7/Ses i 7.0101	11S globulin	AAK15087.1	EGQLIIVPQNYVVAK

This study presents the discovery of unique peptide markers, but further, a large-scale study is needed to confirm their utility in the authentication of commercially manufactured cold-pressed oils. The specificity of the peptide marker should be also further verified using different cultivars, geographical locations, etc. for each species.

## Conclusions

This study provides a set of specific proteins and species-specific peptide markers which can be helpful for food analysts to verify the declared composition of cold-pressed oil as well as the presence of food allergens. Ninety-two specific peptides unique to coconut, evening primrose, flax, hemp, milk thistle, nigella, pumpkin, rapeseed, sesame, and sunflower were detected and identified in cold-pressed oils. The unique peptides were released mainly from specific seed storage proteins and oleosins. Additionally, several food allergens, i.e. pumpkin, sunflower, and sesame allergenic proteins, were observed in the relevant cold-pressed oils. The results are undoubtedly beneficial in the era of widely used unlawful practices, mainly the modification of product composition with cheaper substitutes.

## Methods

### Reagents and samples

Acetonitrile (LC–MS grade) and formic acid (MS grade) were obtained from Sigma-Aldrich (Schnelldorf, Germany). Sequence-grade modified trypsin gold, lyophilized, was obtained from Promega GmbH (Mannheim, Germany). Reversed-phase Sep-Pak C18 Plus cartridges, sorbent weight 360 mg/0.7 mL, were obtained from Waters (Milford, MA, USA). All other chemicals were purchased from Sigma-Aldrich, at the best available purity grade.

The material for the study consisted of 10 selected oilseeds, namely, coconut (*Cocos nucifera* L.), evening primrose (*Oenothera biennis* L.), hemp (*Cannabis sativa* L.), flax (*Linum usitatissimum* L.), milk thistle (*Silybum marianum* L.*)*, nigella/black cumin (*Nigella sativa* or *N. indica*), pumpkin (*Cucurbita pepo* L.), rapeseed (*Brassica napus* L.), sesame (*Sesamum indicum* L.), and sunflower (*Helianthus annuus* L.). The seeds were obtained from the Polish company SemCo Sp. z o.o. (Szamotuły near Poznań) which specializes in the production of oils. Coconut shreds were purchased at a supermarket. Seeds were stored at 4 °C until further proteomic analysis.

### Preparation of samples

The oil was prepared in a cold-pressing process using a Yoda YD-ZY-02A oil press (Warsaw, Poland). The oil temperature during the production process was in the range of 38−50 °C and the efficiency of the pressing process in relation to the oil content was approximately 85%. Protein extraction with acetone was performed according to Martín-Hernández, Bénet, & Obert^15^ with some changes. To 50 g of oil, 125 mL of cold acetone was added. After shaking, the mixture was stirred on a magnetic stirrer at 500 rpm for 1 h at 4 °C. The mixture was then centrifuged at 11,000 RCF and 4 °C for 15 min, and the supernatant was discarded. The precipitate was washed twice with 5 mL of cold acetone. The pellet was dried overnight in drying oven at 40 °C, and then stored at − 20 °C until proteomic analysis. The samples were analysed in duplicate.

### Sodium dodecyl sulphate–polyacrylamide gel electrophoresis (SDS–PAGE)

SDS-PAGE was performed to compare the profiles of the proteins extracted from oil samples according to a previously described method^27^. Dried pellet (5 mg) was dissolved with lysis buffer (8 M urea, 2 M thiourea, 0.05 mM Tris, 75 mM dithiothreitol (DTT), 3% SDS, and 0.05% bromophenol blue, at pH = 6.8) and heated at 98 °C for 4 min. The protein concentration was determined using a 2-D Quant kit (GE Healthcare Bio-Sciences, Fairfield, CT, USA). Protein aliquots (12 μg) were loaded onto 15% polyacrylamide gels prepared in a Hoefer SE250 system (GE Healthcare Bio-Sciences). A reference broad-range molecular weight standard (Bio-Rad Laboratories, Inc., CA, USA) was applied. Gels were run at a constant current of 20 mA per gel, then stained with Coomassie brilliant blue and scanned (Gel Doc XR + System, Bio-Rad Laboratories, Inc., CA, USA).

### In-solution tryptic digestion

Protein digestion was carried out as previously described^26^ with slight modifications. Extracted proteins (3 mg) were rehydrated in 100 μL of 50 mM ammonium bicarbonate. The proteins were reduced by 200 mM DTT (56 °C for 1 h) and then alkylated using 200 mM iodoacetamide for 30 min in the dark at room temperature. The remaining iodoacetamide was quenched by the addition of 200 mM DTT and incubation at room temperature for 30 min. The samples were digested in an ammonium bicarbonate solution containing 0.083 μg/μL trypsin (Promega GmbH, Mannheim, Germany), at 37 °C for overnight. The digests were purified by reversed-phase extraction using Sep-Pak C18 Plus cartridges (Waters, Milford, MA, USA). The SPE column was equilibrated with solvent A consisting of 98% water, 2% acetonitrile, and 0.1% formic acid, then with solvent B consisting of 65% acetonitrile, 35% water, and 0.1% formic acid. The sample (0.6 mL) was then added to the cartridge and washed with solvent A. The peptides were eluted with solvent B and vacuum-dried in a centrifugal evaporator (miVacDuo Concentrator, Genevac Ltd., Suffolk, UK). Samples were resuspended in 2% acetonitrile in Milli-Q water containing 0.1% formic acid (solvent A) before UHPLC-Q-TOF–MS/MS analyses.

### Proteins and peptides identification

Mass spectrometry analysis was according to a previously described procedure^26^ with slight modifications. UHPLC-Q-TOF–MS/MS analysis was performed on an Agilent Technologies 1290 Infinity series liquid chromatograph (Santa Clara, CA, USA) composed of a binary pump, a thermostat, and an autosampler, coupled to a 6550 UHD iFunnel Q-TOF LC/MS. Compounds were ionized by electrospray ionization (ESI) using a JestStream Technology ion source. Chromatographic separation was performed on a 2.1 × 150 mm, 1.8 μm particle-size Agilent RRHD Eclipse Plus C18 column. Instrument control and data acquisition were performed using Agilent MassHunter Workstation Software. The LC parameters were set as follows: 10 μL injection volume, 0.3 mL/min mobile phase flow. The mobile phase consisted of 0.1% formic acid in water (solvent A) and 0.1% formic acid in acetonitrile (solvent B). Gradient steps were applied as follows: 0–2 min, 2% B; 2–40 min, to 32% B; 40–45 min, to 37% B; 45–50 min, to 90% B; 50–55 min, 90% B; and a 5-min post-run at 2% B. The ion source gas (nitrogen) temperature was 250 °C, the flow rate was 14 L/min, nebulizer pressure was 35 *psi*, sheath gas temperature was 250 °C, and sheath gas flow was 11 L/min. The capillary voltage was set at 3500 V, nozzle voltage to 1000 V, and the fragmentor to 400 V. Positive ions formed in an electrospray were acquired in the range of 100–3000 m*/z* in MS scan mode and in auto MS/MS mode, with a scan rate of 5 scan/s for MS and 3 scan/s for MS/MS. Internal mass calibration was enabled by using two reference masses at 121.0509 and 922.0098 m*/z*.

A National Center for Biotechnology Information (NCBI, U.S. National Library of Medicine) protein database search for protein and peptide identification was performed, using the Spectrum Mill MS Proteomics Workbench with > 50% score peak intensity and 10 ppm precursor mass tolerance, with the following parameters: trypsin enzyme, taxonomy green plants or a given taxonomy genus (i.e. *Brassica*, *Cannabis*, *Cocos*, *Cucurbita*, *Helianthus*, *Nigella*, *Linum*, *Oenothera*, *Sesamum*, *Silybum*), two missed cleavages, 50 ppm products mass tolerance, carbamidomethylation as fixed modification, and methionine oxidation as a variable modification. The matches and Spectrum Mill scores were evaluated at 1% of the false discovery rate (FDR), for identity and homology thresholds. Selected peptides, in FASTA format, were searched against the NCBInr database, using the protein Basic Local Alignment Search Tool (BLAST) and blastp algorithm (U.S. National Library of Medicine, Bethesda, MD), for species and protein specificity.

## Supplementary information

Supplementary Information.

Supplementary Information.

## Data Availability

The datasets generated and analysed during this study are available from the corresponding author on reasonable request.
